# Comparison of Plasma-Polymerized Thin Films Deposited from 2-Methyl-2-oxazoline and 2-Ethyl-2-oxazoline: I Film Properties

**DOI:** 10.3390/ijms242417455

**Published:** 2023-12-14

**Authors:** Pavel St’ahel, Věra Mazánková, Lubomír Prokeš, Vilma Buršíková, Monika Stupavská, Marián Lehocký, Hana Pištěková, Kadir Ozaltin, David Trunec

**Affiliations:** 1 Department of Plasma Physics and Technology, Faculty of Science, Masaryk University, Kotlářská 2, 611 37 Brno, Czech Republic; pstahel@physics.muni.cz (P.S.); luboprok@gmail.com (L.P.); vilmab@physics.muni.cz (V.B.); stupavska@mail.muni.cz (M.S.); 2 Department of Mathematics and Physics, Faculty of Military Technology, University of Defence in Brno, Kounicova 65, 662 10 Brno, Czech Republic; vera.mazankova@unob.cz; 3 Centre of Polymer Systems, Tomas Bata University in Zlín, Trida Tomase Bati 5678, 760 01 Zlín, Czech Republicpistekova@utb.cz (H.P.); kadirozaltin@hotmail.com (K.O.)

**Keywords:** antibiofouling, plasma polymer, poly(2-oxazoline)

## Abstract

Poly(2-oxazoline) is a promising new class of polymeric materials due to their antibiofouling properties and good biocompatibility. Poly(2-oxazoline) coatings can be deposited on different substrates via plasma polymerization, which can be more advantageous than other coating methods. The aim of this study is to deposit poly(2-oxazoline) coatings using a surface dielectric barrier discharge burning in nitrogen at atmospheric pressure using 2-methyl-2-oxazoline and 2-ethyl-2-oxazoline vapours as monomers and compare the film properties. For the comparison, the antibacterial and cytocompatibility tests were peformed according to ISO norms. The antibacterial tests showed that all the deposited films were highly active against *Staphylococcus aureus* and *Escherichia coli* bacteria. The chemical composition of the films was studied using FTIR and XPS, and the film surface’s properties were studied using AFM and surface energy measurement. The cytocompatibility tests showed good cytocompatibility of all the deposited films. However, the films deposited from 2-methyl-2-oxazoline exhibit better cytocompatibility. This difference can be explained by the different chemical compositions and surface morphologies of the films deposited from different monomers.

## 1. Introduction

Poly(2-oxazoline)s (POx)s are a unique class of polymers with enormous potential for the preparation of functional materials [1,2,3]. POx coatings are an example of antibiofouling coatings [4,5], which could replace the poly(ethylene glycol) coatings used as hydrophilic antibiofouling coatings now. POxs are promising platforms for drug delivery applications due to their biocompatibility and stealth properties [6]. Plasma-polymerized POx thin films from oxazoline precursors were investigated and optimized as an alternative approach for electrode functionalization for electrochemical immunosensors [7,8]. Currently, there are many applied studies related to the water solubility, invisibility and thermoresponsive of POxs. These studies were focused on the abovementioned applications and on protein modification, gene carriers, cell sheet engineering and hydrogel; see the review [9]. Recent studies include, e.g., an X-ray scattering study of the crystallinity degree of poly(2-methyl-2-oxazoline) [10] or the use of electron beam irradiation as a method to produce non-biofouling POx hydrogel coatings [11].

The formation of POx coatings using conventional methods (e.g., cationic ring-opening polymerization or grafting) is a lengthy and complex multistep wet procedure that can be conducted only on a limited range of substrates. Moreover, the formation of POx coatings via conventional methods creates organic waste. Plasma-based strategies offer several advantages for the polymerization of antibacterial biomaterials and can be used directly or combined with other surface modification techniques [12,13]. Plasma polymerization is a one-step dry process that doesn’t require prior substrate preparation and eliminates the use of organic solvents. Recently, plasma polymerization of 2-oxazolines was used to produce robustly attached coatings on different substrates. The plasma polymerization of POx from 2-ethyl-2-oxazoline was performed in a low-pressure inductively excited pulsed radio frequency (RF) discharge [14]. Later, plasma deposition of 2-methyl-2-oxazoline and 2-ethyl-2-oxazoline was carried out in a low-pressure RF discharge [15,16,17,18].

In our previous studies, POx coatings were deposited on glass substrates using plasma polymerization with 2-methyl-2-oxazoline or 2-ethyl-2-oxazoline vapours as a monomer [19,20]. The plasma polymerization was performed in a volume dielectric barrier discharge (DBD) burning in nitrogen at atmospheric pressure. The coatings deposited from 2-methyl-2-oxazoline exhibited better antibacterial activity against *E. coli* and *S. epidermidis* bacterial strains and exhibited better cytocompatibility. The best antibacterial film properties and cytocompatibility were achieved by increasing the substrate temperature at the deposition. However, the deposition of the antibacterial coating on polymeric materials must be conducted at low temperatures only due to their sensitivity to higher temperatures. Preliminary experiments showed that POx deposition on polycaprolactone nanofibers was impossible in a volume DBD because these nanofibers were melting. So, for further experiments with POx coating deposition, surface DBD was chosen because this discharge shortens the deposition time and decreases the gas temperature in the discharge. Also, teflon (PTFE) as a substrate was chosen as a polymeric material—one of the most thermally stable.

In this study, two series of POx coatings were deposited on teflon substrates—the first series using 2-methyl-2-oxazoline vapours as a monomer and the second series using 2-ethyl-2-oxazoline vapours as a monomer. The plasma polymerization was performed in atmospheric pressure surface DBD burning in nitrogen. The same discharge had already been used for the plasma polymerization of thin films from a propane–butane mixture [21]. Using this discharge shortened the deposition time from 23 min (in volume DBD) to 1.5 min. The deposited POx films were analyzed using FTIR and XPS, and the morphology of the films was studied using AFM. Also, antibacterial and cytocompatibility tests were performed.

## 2. Results

### 2.1. Antibacterial Properties

The antibacterial tests were performed according to ISO 22196:2011 [22] using *S. aureus* (CCM 4516) and *E. coli* (CCM 4517) bacteria. The results of the number of viable bacteria per cm${}^{2}$ of a sample (CFU/cm${}^{2}$) are given in Table 1 for films deposited from 2-methyl-2-oxazoline and 2-ethyl-2-oxazoline monomers. These numbers are the arithmetic mean of CFU/cm${}^{2}$ from three samples. Most of the samples showed very strong antibacterial activity against both bacterial strains, except for the last sample in Table 1. Only significant antibacterial activity against *E. coli* was measured for this sample, and there was no antibacterial activity against *S. aureus*.

### 2.2. Cytocompatibility Results

**Figure 1:**
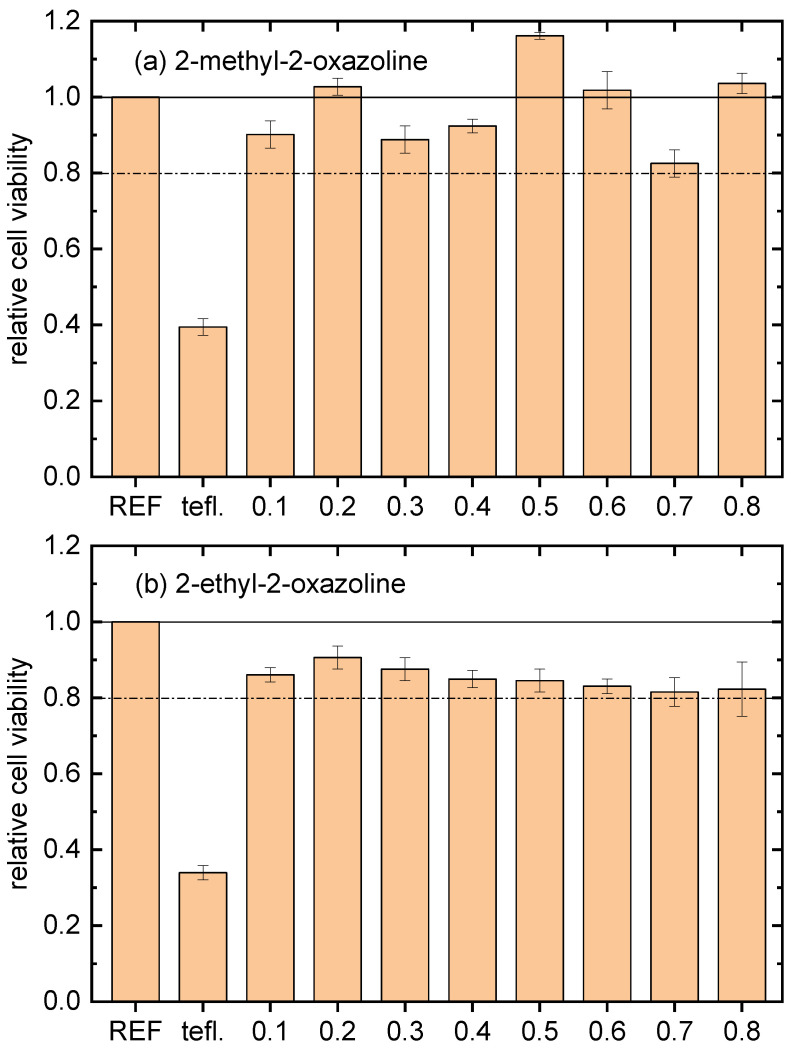
In vitro cytocompatibility results of tested POx films deposited at different monomer flow rates. REF marks polystyrene tissue, tefl. marks bare teflon substrate and the numbers are nitrogen flow rates through the corresponding monomer in slm. The horizontal black dashed line marks 80% of the cell viability threshold compared with the reference polystyrene tissue. (**a**) Coatings deposited from 2-methyl-2-oxazoline; (**b**) coatings deposited from 2-ethyl-2-oxazoline.

In vitro cytocompatibility results were obtained using mouse embryonic fibroblast (NIH/3T3) cells. The duration of the cell interaction with the tested samples was 48 h. The results obtained are presented in Figure 1, where cell viability for the bare teflon and the film-coated counterparts is presented in comparison with the reference polystyrene tissue. The values shown in Figure 1 are the arithmetic means with the standard deviations from three samples. The cytocompatibility of all the POx coatings was higher than 80% of the cell viability threshold compared with the reference polystyrene tissue. This result showed that all the deposited films are cytocompatible, besides performing antibacterial properties. Moreover, the films deposited from 2-methyl-2-oxazoline at nitrogen flow through a monomer of 0.2, 0.5, 0.6 and 0.8 slm have better cytocompatibility than polystyrene tissue and the films deposited from 2-ethyl-2-oxazoline. The best cytocompatibility was observed at the film deposited at a monomer flow rate of 0.5 slm. So, this film and the film deposited from 2-ethyl-2-oxazoline at the same monomer flow rate were further analyzed using AFM, FTIR and XPS.

### 2.3. Surface Characterization

**Figure 2:**
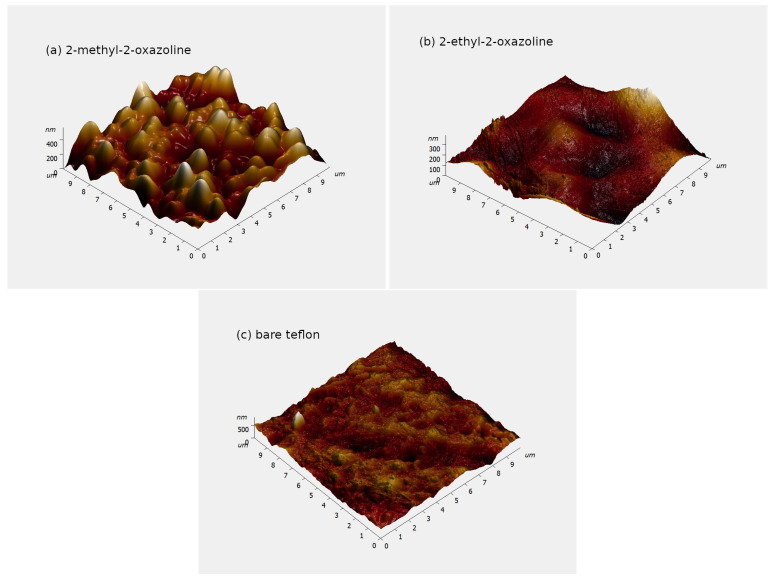
Atomic Force Microscope (AFM) images of POx films. (**a**) Deposited from 2-methyl-2-oxazoline; (**b**) deposited from 2-ethyl-2-oxazoline; (**c**) bare teflon.

The AFM images of POx films and bare teflon are shown in Figure 2. As it can be seen, the coating deposited from 2-methyl-2-oxazoline has the highest roughness, the coating deposited from 2-ethyl-2-oxazoline has the lowest roughness, and even the roughness of bare teflon is smoothed out by this coating. The range of the *z*-axis (surface height) in these graphs was set by NovaPx software v3.4 according to the highest value in the AFM scan. A single protrusion can expand the *z*-axis range, as happened in the case of bare teflon. The surface roughness is properly described by the root mean square (RMS) roughness and average roughness.

The root mean square roughness and the average roughness of POx films and bare teflon are given in Table 2. These roughnesses were determined at five places with dimensions 10μm×10μm at each sample and were averaged.

The film thickness was estimated using a depth-sensing indentation technique. The thickness of the film deposited from 2-methyl-2-oxazoline was 600 nm and the thickness of the film deposited from 2-ethyl-2-oxazoline was 750 nm. Both types of coatings were partially soluble in water, as was observed in previous studies [19,20].

The contact angles between the test liquids and POx films were measured using the sessile drop technique in order to determine the total surface free energy. Three test liquids (distilled water, glycerol and diiodomethane (CH${}_{2}$I${}_{2}$)) were used for this measurement, and the acid–base theory, with multiple regression [23], was used to calculate the total surface free energy. The deposited films were hydrophilic with the water contact angle in the range of 15–23° and the surface free energy in the range of 42–65 mJ/m${}^{2}$.

**Figure 3:**
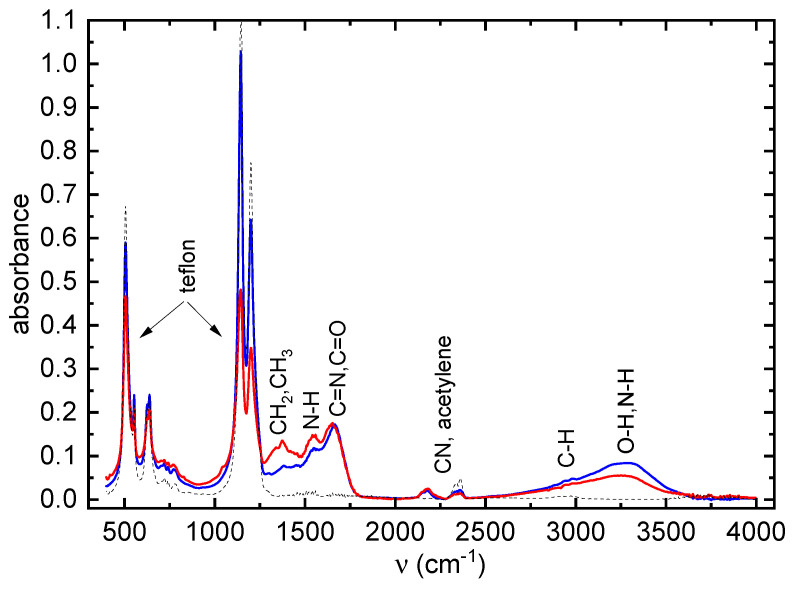
FTIR spectra of POx thin films. 2-ethyl-2-oxazoline coating—blue line; 2-methyl-2-oxazoline layers—red line; bare teflon—black dashed line.

The FTIR spectra of the deposited POx thin films are shown in Figure 3.

A broad absorption band in the range 3000–3600 cm${}^{-1}$ consists of several peaks belonging to stretching vibrations of OH, NH and NH${}_{2}$ groups. The bands between cca 1460 and 1350 cm${}^{-1}$ are characteristic of the bending vibrations of CH${}_{3}$ and CH${}_{2}$ groups, although the bands of stretching vibrations of these groups (cca 3000–2500 cm${}^{-1}$) are weak. The band at 2170 cm${}^{-1}$ can be attributed to nitrile C≡N and/or isocyanate O=C=N groups. The band between 1800 cm${}^{-1}$ and 1600 cm${}^{-1}$ is typical for the stretching vibration of C=N (constituting the oxazoline ring) and/or a C=O bond. Its presence in the IR spectrum indicates the presence of oxazoline rings in deposited films. The band around 1550 cm${}^{-1}$ belongs to N-H bonds. Finally, the bands below 1350 cm${}^{-1}$ belong to teflon substrate. No significant difference was observed between the FTIR spectra of the 2-methyl- and 2-ethyl-2-oxazoline layers.

The atomic concentrations of all the elements presented on the surface of the studied samples obtained from XPS measurement are summarized in Table 3. As expected for a PTFE polymer, the untreated surface contained mostly carbon (42%), fluorine (54%) and oxygen (4%). A small amount of oxygen observed on the untreated PTFE surface originates from the adventitious contamination layer adsorbed on the polymer surface.

**Figure 4:**
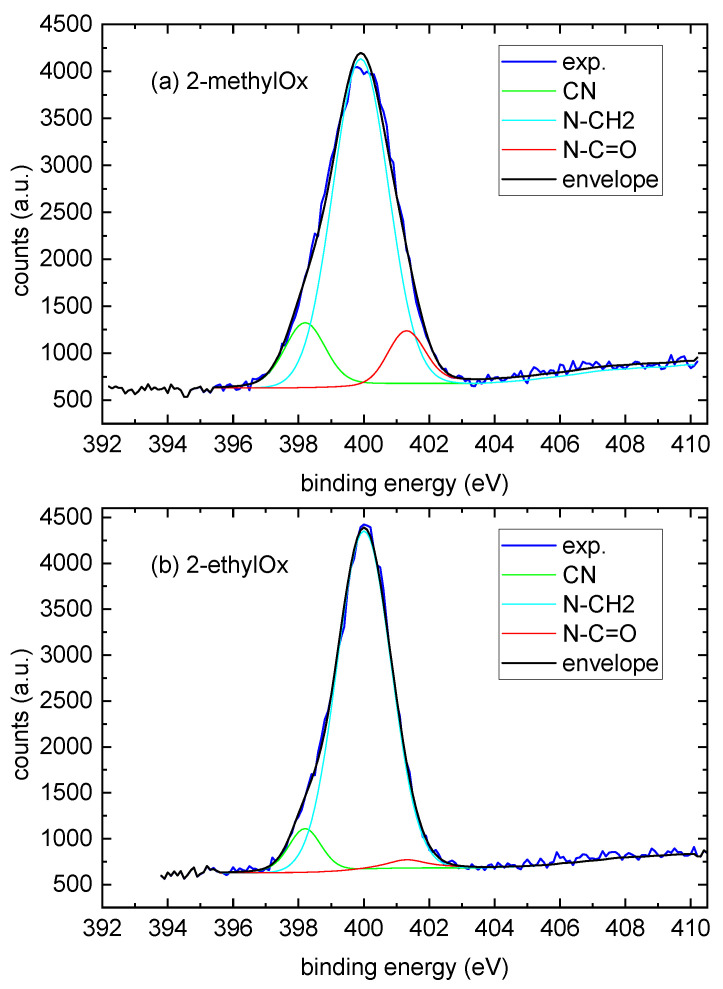
Curve fitting analysis of the high-resolution XPS N1s spectrum of POx films. (**a**) Deposited from 2-methyl-2-oxazoline; (**b**) deposited from 2-ethyl-2-oxazoline. Exp. marks the result from XPS measurement; envelope marks the sum of deconvoluted components.

The deposited POx coatings contain, besides carbon, a large amount of nitrogen. There is a large difference in the nitrogen content between both types of coatings (24% for coatings deposited from 2-methyl-2-oxazoline and 15% for coatings deposited from 2-ethyl-2-oxazoline). Also, oxygen is present in the deposited coatings, though the difference in oxygen content between both types of coating is not so significant. To evaluate which functional groups are formed in the deposited coatings, the C1s and N1s high-resolution spectra were investigated in detail. The C1s peak of the reference teflon sample was deconvoluted into four components. The components at binding energies 284.9 eV, 286.2 eV, 289.1 eV and 292.20 eV were attributed to C-C/C-H, C-O, O-C=O and C-F2 bonds. Deconvolution of the C1s peak of POx films showed the formation of new chemical bonds containing nitrogen (C-N, C≡N, N-C=O) and oxygen (C=O). The results of the C1s peak deconvolution are shown again in Table 3. For the coatings deposited from 2-methyl-2-oxazoline, the concentration of C-N and N-C=O chemical bonds reached 22% and 21%, respectively. In coatings deposited from 2-ethyl-2-oxazoline, the higher concentration within nitrogen-containing groups had a C-N chemical bond. The N1s peaks of the deposited POx coatings were deconvoluted into three components. The components at binding energies 398.2 eV, 399.9 eV and 401.3 eV were attributed to C≡N, H${}_{2}$N-C and N-C=O bonds; see Figure 4.

The results of the N1s peak deconvolution are shown again in Table 3. The concentration of N≡C bonds is markedly higher for coatings deposited from 2-methyl-2-oxazoline. The same trend was observed as well for the N-C=O chemical bond, where the concentration for the coatings deposited from 2-ethyl-2-oxazoline was six times lower than with the coatings deposited from 2-methyl-2-oxazoline. This is in good agreement with the C1s results, which showed the presence of nitrogen containing thet chemical bonds C-N, C≡N and N-C=O. The XPS results are consistent with the conclusions obtained via FTIR spectroscopy.

## 3. Discussion

The plasma-polymerized POx coatings were deposited on teflon substrates using 2-methyl-2-oxazoline and 2-ethyl-2-oxazoline vapours as monomers using a surface DBD burning in nitrogen. The use of a surface DBD allowed for the shortening of the deposition time compared with the deposition time in a volume DBD. The deposited films exhibited very strong antibacterial activity against bacterial strains—namely, gram-negative *E. coli* and gram-positive *S. aureus*. The exact antibacterial mechanism of action of 2-methyl-2-oxazoline and 2-ethyl-2-oxazoline on bacteria is still a subject of research. Nevertheless, the antibacterial mechanism of oxazolines was described in detail by Dai et al. [24]. They discovered that poly(2-oxazoline)s bind to intracellular targets, such as DNA, RNA and proteins, and disrupt the metabolic pathways and cellular processes of bacteria [24,25]. Compared with the reference polystyrene tissue, the POx films were cytocompatible, reaching more than 80% of the cell viability threshold. The films deposited from 2-methyl-2-oxazoline had better cytocompatibility than the films deposited from 2-ethyl-2-oxazoline, and their cytocompatibility was, for some values of monomer flow, even better than the cytocompatibility of the reference polystyrene tissue. This can be caused by the higher nitrogen content in the films deposited from 2-methyl-2-oxazoline, because nitrogen-rich surfaces are known for their excellent biocompatibility [26]. Also, the surface roughness of the 2-methyl-2-oxazoline deposited films is higher, which can lead to slightly greater cell growth. So, using 2-methyl-2-oxazoline as a monomer for POx film deposition is more useful than using 2-ethyl-2-oxazoline. Furthermore, the use of nitrogen as a working gas in a DBD enables the obtaining of POx films with higher nitrogen content.

Our next study will be focused on the detailed analysis of the deposition process which could contribute to a better understanding of the chemical composition and antibacterial activity of deposited POx films. Further, the depositions of antibacterial coatings on other polymers, e.g., polypropylene, are in progress.

## 4. Materials and Methods

### 4.1. Materials

Polytetrafluoroethylene (PTFE, teflon) foils (TFP universal a.s., Dobřejovice, Czech Republic) with dimensions of 130 mm × 400 mm and thickness 1 mm were used as substrates for deposition. 2-Methyl-2-oxazoline and 2-ethyl-2-oxazoline (Sigma-Aldrich, Munich, Germany) were used as monomers for plasma deposition. The antibacterial tests were performed with *S. aureus* (CCM 4516) and *E. coli* (CCM 4517), both supplied by the Czech Collection of Microorganisms in Brno. These bacterial cultures were grown into commercial BHI medium (Brain Heart Infusion Broth, HiMedia, Mumbai, India). Basic Red 2 (Safranin O, Sigma-Aldrich, Munich, Germany) stain was used for biofilm visualization.

### 4.2. Plasma Deposition

Plasma polymerization of POx thin films was performed in a custom-built reactor with surface dielectric barrier discharge (SDBD) which was described in detail in our previous paper [21]. The discharge burned at atmospheric pressure in nitrogen with an admixture of monomer vapour. The total gas flow through the discharge was set to 5.4 slm and it was mixed from two flows. The first pure nitrogen flow had flow rates from 5.3 slm to 4.6 slm and the second nitrogen flow had flow rates from 0.1 slm to 0.8 slm and bubbled through the liquid 2-methyl-2-oxazoline or 2-ethyl-2-oxazoline monomer in a glass bottle container. The temperature of the monomer was kept constant and set to 20 ${}^{\circ }$C. The applied discharge voltage was 6 kV and the discharge power was set to 140 W. The teflon substrate was periodically moved between the upper electrodes and lower electrode of SDBD with a speed of 14.5 cm min${}^{-1}$. The deposition time was 4 min for nitrogen flow 0.1 slm through the monomer and the deposition time linearly decreased up to 1.5 min for nitrogen flow 0.8 slm through the monomer. This adjustment of deposition times ensures approximately the same film thickness at all monomer flow rates.

### 4.3. Surface Characterization

The IR spectra of deposited films were measured using FTIR spectrometer Alpha (Bruker, Billerica, MA, USA) using an ATR module Platinum (diamond ATR-crystal, single reflection, monolithic, edged in tungsten carbide). The total surface free energy of the films was determined from measurements of contact angles between testing liquids and the film surfaces using a sessile drop technique. The acid-base theory was used for the calculation of total surface free energy.

Atomic Force Microscope (AFM) Ntegra Prima (NT-MDT, Apeldoorn, The Netherlands) was used to study the surface topography of the POx films. The measurements were performed in semi-contact mode on 10μm×10μm and 20μm×20μm areas of each coating with a scanning rate of 0.5 Hz. The 3D roughness parameters of the thin films were evaluated according to the ASME B46 standard using the NovaPx software v3.4 (NDMDT).

The XPS measurements were performed on an ESCALAB 250Xi (Thermo Fisher Scientific, East Grinstead, UK). An X-ray beam with a power of 200 W (650 microns spot size) was used. The survey spectra were acquired with a pass energy of 50 eV and an energy step of 1 eV. High-resolution scans were acquired with a pass energy of 20 eV and an energy step of 0.1 eV. In order to compensate for charges on the surface, an electron flood gun was used. Spectra were referenced to the hydrocarbon type C1s component set at a binding energy of 284.8 eV. Spectra calibration, processing and fitting routines were performed using Avantage software v5.9925.

### 4.4. Antibacterial Tests

Before conducting antibacterial testing, samples underwent disinfection using UV radiation. Polypropylene foils employed in the tests were disinfected through rinsing with 70% denatured ethanol. Two bacterial strains, namely gram-negative *E. coli* (CCM 4517) and gram-positive *S. aureus* (CCM 4516), were utilized for the tests. The antibacterial testing followed the ISO 22196 [22] protocol with certain modifications. Bacterial suspensions (*E. coli* $3.1\times 10^{5}$ CFU mL${}^{-1}$; *S. aureus* $5.1\times 10^{5}$ CFU mL${}^{-1}$) were prepared in 1/500 nutrient broth (HiMedia laboratories, Mumbai, India). Nutrient broth consisted of peptone (10 g L${}^{-1}$), beef extract (10 g L${}^{-1}$), sodium chloride (5 g L${}^{-1}$, pH 7.3±0.1). Nutrient broth was diluted with deionized water to a 500-fold volume, and the pH was adjusted to 7.0±0.1 before sterilization via autoclaving. A 100 μL volume of the bacterial suspension was dispensed onto the sample surface (dimensions 25 mm × 25 mm), which was then covered with a polypropylene foil (dimensions 20 mm × 20 mm). The samples with foils were cultivated at 35 ${}^{\circ }$C and 100% relative humidity for 24 h. After the incubation time, the polypropylene foil was removed and each sample underwent thorough washing with SCDLP broth (HiMedia laboratories, Mumbai, India), which was subsequently collected. The viable bacteria count was determined using the pour plate culture method (PCA, HiMedia laboratories, Mumbai, India). All tests were performed in triplicate.

### 4.5. Cytocompatibility Test

The mouse embryonic fibroblast continuous cell line (NIH/3T3, ATCC^®^ CRL-1658™, Teddington, UK) was utilized for cytocompatibility testing following the EN ISO 10993-5 standard [27], with modification. The culture medium used was the ATCC-formulated Dulbecco’s Modified Eagle’s Medium (BioSera, Nuaille, France), which included 10% calf serum (BioSera, France) and Penicillin/Streptomycin at 100 U mL${}^{-1}$ (PAA Laboratories GmbH, Pasching, Austria). The tested samples, with a dimension of 10 mm × 10 mm, were sterilized via UV–radiation (wavelength of 258 nm) for 30 min and placed into a 24-well plate. The cells were seeded onto the samples at a concentration of $1\times 10^{4}$ for one hour to facilitate cell adhesion. Following pre-cultivation, a sufficient amount of the medium was added, and the samples were incubated for 48 h at 37 ${}^{\circ }$C. The changes in cell morphology were observed using an inverted fluorescent microscope (Olympus, IX 81). To assess the cytotoxic effects, an MTT assay (Duchefa, Biochemie, Haarlem, The Netherlands) was conducted and absorbance was measured using an Infinite M200 Pro NanoQuant absorbance reader (Tecan, Männedorf, Switzerland). All tests were performed in triplicate.

## Figures and Tables

**Table 1:** Antibacterial activity results of studied POx films. MeOx marks 2-methyl-2-oxazoline, EtOx marks 2-ethyl-2-oxazoline and none mark bare teflon substrate. Nitrogen flow is the flow through corresponding monomer; total nitrogen flow through the discharge was set to 5.4 slm.

Monomer	Nitrogen Flow (slm)	*S. aureus* CCM 2022 (CFU/cm${}^{2}$)	*E. coli* CCM 4517 (CFU/cm${}^{2}$)
None		$4.1\times 10^{5}$	$1.4\times 10^{6}$
MeOx	0.8	8.1	$7.7\times 10^{2}$
MeOx	0.7	6.3	$1.4\times 10^{3}$
MeOx	0.6	53	$1.8\times 10^{3}$
MeOx	0.5	57	$7.8\times 10^{2}$
MeOx	0.4	33	$2.4\times 10^{3}$
MeOx	0.3	5.3	$4.9\times 10^{2}$
MeOx	0.2	2.2	$3.7\times 10^{2}$
MeOx	0.1	$4.3\times 10^{2}$	$2.1\times 10^{1}$
EtOx	0.8	<1	$5.8\times 10^{1}$
EtOx	0.7	<1	$2.9\times 10^{1}$
EtOx	0.6	<1	$2.9\times 10^{1}$
EtOx	0.5	<1	$2.3\times 10^{2}$
EtOx	0.4	$1.1\times 10^{3}$	$1.2\times 10^{2}$
EtOx	0.3	<1	$1.5\times 10^{2}$
EtOx	0.2	12	$6.8\times 10^{1}$
EtOx	0.1	$4.3\times 10^{5}$	$8.8\times 10^{3}$

**Table 2:** Root mean square (RMS) roughness and the average roughness of POx films.

Film	RMS Roughness (nm)	Average Roughness (nm)
Bare teflon	80 ± 10	60 ± 10
2-ethyl-2-oxazoline	37 ± 5	29 ± 5
2-methyl-2-oxazoline	130 ± 10	110 ± 10

**Table 3:** Elemental composition and concentration of chemical bonds obtained from deconvolution of C1s and N1s peaks.

Peak	Element or Bond	Teflon	2-Methyl-2-oxazoline	2-Ethyl-2-oxazoline
C1s	F	54	8	10
	C	42	53	62
	O	4	15	13
	N	0	24	15
	O/C	0.095	0.283	0.2
C1s	C-C	49	18	29
	C-N	0	21	34
	C=O	0	4	9
	C-O	4	19	10
	COO	1	13	3
	N-C=O	0	20	10
	C-F	0	0	1
	C-F2	46	1	2
	C-F3	0	2	1
	C≡N	0	2	1
N1s	C≡N	0	11	8
	H${}_{2}$N-C	0	77	90
	N-C=O	0	12	2

## Data Availability

The data presented in this study are available upon reasonable request from the corresponding author.
